# A Robust Optical Sensor for Remote Multi-Species Detection Combining Frequency-Division Multiplexing and Normalized Wavelength Modulation Spectroscopy

**DOI:** 10.3390/s21041073

**Published:** 2021-02-04

**Authors:** Wenling Jin, Hui Zhang, Mai Hu, Mengpeng Hu, Yubin Wei, Jingqiu Liang, Ruifeng Kan, Qiang Wang

**Affiliations:** 1 State Key Laboratory of Applied Optics, Changchun Institute of Optics, Fine Mechanics and Physics, Chinese Academy of Sciences, Changchun 130033, China; jinwenling17@mails.ucas.ac.cn (W.J.); zhanghui195@mails.ucas.ac.cn (H.Z.); humai@ciomp.ac.cn (M.H.); humengpeng19@mails.ucas.ac.cn (M.H.); liangjq@ciomp.ac.cn (J.L.); rfkan@ciomp.ac.cn (R.K.); 2 University of Chinese Academy of Sciences, Beijing 100049, China; 3 Institute of Laser, Shandong Academy of Sciences, Jinan 250014, China; wyb9806@qlu.edu.cn

**Keywords:** multi-species sensor, normalized wavelength modulation spectroscopy, frequency-division multiplexing, remote sensing

## Abstract

By combining frequency-division multiplexing and normalized wavelength modulation spectroscopy, a robust remote multi-species sensor was developed and demonstrated for practical hydrocarbon monitoring. Independently modulated laser beams are combined to simultaneously interrogate different gas samples using an open-ended centimeter-size multipass cell. Gas species of interest are demodulated with the second harmonics to enhance sensitivity, and high immunity to laser power variation is achieved by normalizing to the corresponding first harmonics. Performance of the optical sensor was experimentally evaluated using methane (CH_4_) and acetylene (C_2_H_2_) samples, which were separated by a 3-km fiber cable from the laser source. Sub-ppm sensitivity with 1-s time resolution was achieved for both gas species. Moreover, even with large laser intensity fluctuations ranging from 0 to 6 dB, the noise can be kept within 1.38 times as much as that of a stable intensity case. The reported spectroscopic technique would provide a promising optical sensor for remote monitoring of multi hazardous gases with high robustness.

## 1. Introduction

Gas monitoring is crucial for safe operation at chemical plants, coal mines, and gas stations, where leaked toxic, flammable, or explosive gases may cause serious accidents [1,2]. Besides, the ability of simultaneous multiple components detection is attracting rising attention in human breath diagnosis [3]. Real-time identification and quantification of different gases of interest are challenging to bridge the potential danger and an early warning. Among numerous metrologies with electrochemistry [4], Fourier Transform Spectrometer [5], etc., laser absorption spectroscopy has proven to be a promising gas analysis method for high selectivity, high sensitivity, and remote sensing [6,7]. Various typical multi-species sensing techniques were afterwards developed based on photoacoustic spectroscopy (PAS), dual-frequency comb (DFC), and laser heterodyne radiometer (LHR) [8,9,10,11].

To perform multi-species detection, two requirements should be satisfied. Firstly, gas molecules with distinguishable fingerprint spectra can be effectively illuminated by the light source. Secondly, different absorption features can be separately demodulated without any cross interference. Over past decades, multi-species detection based on laser absorption spectroscopy has been well developed. Given that transitions of different gas species may lay beyond the spectral range of a single laser, an approach with a light source comprising multiple lasers is commonly used. Wu et al. reported a dual-gas quartz enhanced photoacoustic spectroscopy (QEPAS) sensor for H_2_O and C_2_H_2_ with two near-infrared lasers, and the excited acoustic signals were separated by two different demodulation frequencies, i.e., the fundamental and the first overtone vibrations of the same QTF [8]. Similarly, Liu et al. employed three diode lasers to demonstrate another H_2_O, CH_4_, and CO_2_ sensor, whose acoustic signals were generated with three resonators and separated at different frequencies [9]. With the development of a supercontinuum light source and diode laser with an external cavity (ECDL), even a single laser is currently capable of covering the selected absorption lines of the target gas species. Rieker et al. reported remote multi-species sensing with DFC, covering a spectral range from 5990 to 6260 cm^−1^, and demonstrated simultaneous measurement of CO_2_, CH_4_, H_2_O, HDO, and ^13^CO_2_ over a 2 km atmospheric path [10]. Differently, Wang et al. utilized the sunlight as the light source to interrogate the earth’s atmosphere, of which the CO_2_ and CH_4_ column-averaged abundances were simultaneously acquired by two local oscillators using LHR [11]. However, among most of the above typical techniques, the sensors’ performance, especially non-linear spectroscopy, e.g., PAS-based sensors, replies critically on the stability of light power. A stable source power is needed in the gas sensing implementation, otherwise false measurements in practical implementation would appear.

Many efforts are contributed in the optical sensing society to overcome the issues. Constant laser output power over a laser-frequency scan has been achieved by an active servo loop with an acousto-optic modulator (AOM) [12] or an electro-optic modulator (EOM) [13]. This offers an advantage of simplifying enormously the spectral analysis. Besides, influence of laser fluctuation on the spectrum recovery can also be effectively suppressed by a dual-beam regime with balanced detection [14] or a division process [15]. However, for both single-beam and dual-beam regimes, it is still challenging to stabilize the light power coupled on photodetectors, especially for remote sensing after long-distance transmission with inevitable and irregular scattering loss, inconstant transmission loss, even devices’ unstable operation. On the contrary, a calibration-free wavelength modulation spectroscopy was proposed for recovering the absorption profile from harsh environments [16,17], by which the unstable laser influences in single-beam regimes could be post processed with careful laser modulation characterization.

In this work, we demonstrated a robust sensor with high immunity to lasers’ power noise. Frequency-division multiplexing (FDM) and normalized wavelength modulation spectroscopy are harnessed towards real-time remote multi-species sensing with simpler analysis. Different gas species of interest are simultaneously interacted by the same open-ended probe with individually modulated fiber-coupler diode lasers, which are combined before interaction and the subsequent coupling on one single photodetector. The target gases are demodulated based on FDM with separate digital lock-in amplifiers (LIA) on a LabVIEW platform. The robustness is enhanced by normalizing to the corresponding first harmonic signals, deriving from the same time-domain signal, to remove the influence of irregular laser intensity fluctuation. With simultaneous C_2_H_2_ and CH_4_ measurements at a harsh operation condition, the sensor’s characterization and performance were experimentally investigated. Besides, only the non-electrified probe serves in the work area while all the others could operate in a much gentler monitoring area, guaranteeing intrinsic safety and stable operation for hazardous gas sensing.

## 2. Principle

The transmission coefficient *τ*(*ν*) of laser radiation through a medium length of *L* is governed by Beer-Lambert law, which relates the transmitted intensity *I_t_* to the incident intensity *I*_0_ as [18]

(1) $$\tau \left(\nu \right)=\left(\frac{I_{t}}{I_{0}}\right)=\exp\left[-\alpha \left(\nu \right)\right]\approx 1-P\chi_{i}L\sum_{j}S_{j}\left(T\right)\varphi_{j}\left(\nu \right)$$

where *α*(*ν*) represents the spectral absorbance at optical frequency *ν*, *P* is the total gas pressure, *χ_i_* is the mole fraction of the target specie, *S_j_*(*T*) and *φ_j_*(*ν*) are the line strength and line-shape function of *j*th absorption feature respectively. The summation accounts for the overlap of adjacent absorption features, which is variable by collisional broadening at different pressures. With wavelength modulation at a frequency of *ω* = 2π*f* via the laser injection current, the laser intensity *I*_0_ is simultaneously modulated but with a WM/IM phase shift and an instantaneous nonlinear response [19,20].

(2) $$\nu \left(t\right)=\bar{\nu }+a\cos\left(\omega t\right)$$

(3) $$I_{0}(t)={\bar{I}}_{0}[1+\underset{\begin{array}{cc} linear & part \end{array}}{\underset{︸}{i_{0}\cos(\omega t+\psi_{1})}}+\underset{\begin{array}{cc} nonlinear & part \end{array}}{\underset{︸}{i_{2}\cos(2\omega t+\psi_{2})}}]$$

where $\bar{\nu }$ is the center frequency, *a* is the modulation depth, ${\bar{I}}_{0}$ is the average laser intensity at $\bar{\nu }$, *i*_0_ and *ψ*_1_ are the intensity modulation amplitude and phase difference of the linear part, *i*_2_ and *ψ*_2_ is the intensity modulation amplitude and phase difference of the nonlinear part. While the spectral absorption can be expanded in a Fourier cosine series as

(4) $$\tau \left[\bar{\nu }+a\cos\left(\omega t\right)\right]=\sum_{k=0}^{k=+\infty }H_{k}(\bar{\nu },a)\cos(k\omega t)$$

where the functions *H_k_*($\bar{\nu }$, a) are given by

(5) $$H_{0}(\bar{\nu },a)=\frac{1}{2\pi }\int_{-\pi }^{+\pi }\left[1-P\chi_{i}L\sum_{j}S_{j}\left(T\right)\varphi_{j}\left(\bar{\nu }+a\cos u\right)\right]du$$

(6) $$H_{k}\left(\bar{\nu },a\right)=\frac{1}{\pi }\int_{-\pi }^{+\pi }\left[1-P\chi_{i}L\sum_{j}S_{j}\left(T\right)\varphi_{j}\left(\bar{\nu }+a\cos u\right)\right]\cos kudu\begin{array}{cc} , & k>0 \end{array}$$

The second harmonic component at 2*f*, extracted from the original detector signal with an LIA, is commonly used to perform absorption-based gas sensing. Combining Equations (1)–(6), the absolute magnitude of the 2*f* signal is given by

(7) $$\begin{array}{lll} R_{2f} & = & \sqrt{{\left(X_{2f}\right)}^{2}+{\left(Y_{2f}\right)}^{2}}\\ & = & \frac{G{\bar{I}}_{0}}{2}\sqrt{\begin{array}{l} {}[H_{2}+\frac{i_{0}}{2}(H_{1}+H_{3})\cos\psi_{1}+i_{2}{\left[(H_{0}+\frac{H_{4}}{2})\cos\psi_{2}\right]}^{2}\\ +[\frac{i_{0}}{2}(H_{1}-H_{3})\sin\psi_{1}+i_{2}(H_{0}-\frac{H_{4}}{2})\sin\psi_{2}){]}^{2} \end{array}} \end{array}$$

where *G* is the equivalent gain factor of the detection system, *X*_2*f*_ and *Y*_2*f*_ are the orthogonal components of the second harmonic signal respectively. The demodulated 2*f* signal is proportional not only to the product of gain factor and the absorption part, which is expressed by the square root function in Equation (7), but also to the laser intensity coupled on the photodetector. Therefore, the stability of ${\bar{I}}_{0}$ is important to the sensor performance, resulting in a potential risk of destroying the reliability of most absorption-based monitoring systems without reasonable compensation algorithms or stability strategies [8,9,10,11,21,22,23]. The issue could be more critical to scenarios where the laser intensity is very sensitive to the practical environment after a long-path transmission via free-space optics [24] or conductive fiber [25].

Similar to Equation (7), the absolute magnitude of the first harmonic signal, extracted from the same time-domain detector signal, can be expressed by

(8) $$\begin{array}{lll} R_{1f} & = & \sqrt{{\left(X_{1f}\right)}^{2}+{\left(Y_{1f}\right)}^{2}}\\ & = & \frac{G{\bar{I}}_{0}}{2}\sqrt{\begin{array}{l} {\left[H_{1}+i_{0}(H_{0}+\frac{H_{2}}{2})\cos\psi_{1}+\frac{i_{2}}{2}(H_{1}+H_{3})\cos\psi_{2}\right]}^{2}\\ +{\left[i_{0}(H_{0}-\frac{H_{2}}{2})\sin\psi_{1}+\frac{i_{2}}{2}(H_{1}-H_{3})\sin\psi_{2}\right]}^{2} \end{array}} \end{array}$$

where *X*_1*f*_ and *Y*_1*f*_ are the orthogonal components of the first harmonic signal respectively. In the architecture of wavelength modulation spectroscopy, 1*f* detection is not preferred for gas demodulation due to distorted waveform with a strong background deriving from residual amplitude modulation (RAM) [6,7]. As analyzed in Equation (8), 1*f* signal is also proportional to the laser intensity ${\bar{I}}_{0}$, because of which *R*_1*f*_ would be a candidate to improve the robustness of absorption-based sensors by counteracting both instantaneous and long-term laser intensity variations coupled on the photodetector. The process is described as normalization using different harmonics from the same photodetector signal, which is shown as

(9) $$\begin{array}{l} S=R_{2f}/R_{1f}=\\ \sqrt{\frac{{\left[H_{2}+\frac{i_{0}}{2}(H_{1}+H_{3})\cos\psi_{1}+i_{2}{\left[(H_{0}+\frac{H_{4}}{2})\cos\psi_{2}\right]}^{2}+[\frac{i_{0}}{2}(H_{1}-H_{3})\sin\psi_{1}+i_{2}(H_{0}-\frac{H_{4}}{2})\sin\psi_{2})\right]}^{2}}{{\left[H_{1}+i_{0}(H_{0}+\frac{H_{2}}{2})\cos\psi_{1}+\frac{i_{2}}{2}(H_{1}+H_{3})\cos\psi_{2}\right]}^{2}+{\left[i_{0}(H_{0}-\frac{H_{2}}{2})\sin\psi_{1}+\frac{i_{2}}{2}(H_{1}-H_{3})\sin\psi_{2}\right]}^{2}}} \end{array}$$

It can be seen that the normalized wavelength modulation signal is independent of the laser intensity coupled on the photodetector. This characteristic makes it a promising approach to a promoted absorption-based gas sensor immune to unexpected intensity variation before/during/after the interaction.

**Figure 1:**
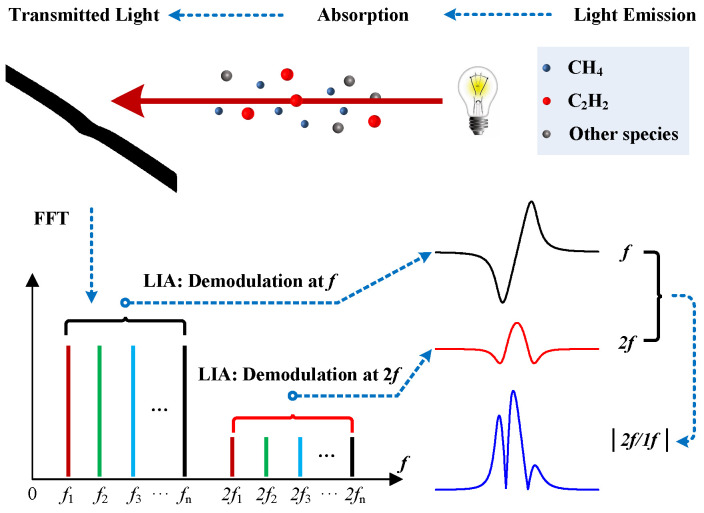
Schematic of the multi-species sensor combining normalized wavelength modulation spectroscopy and frequency-division multiplexing. *f*_1_, *f*_2_ … *f*_n_ equal the possible different modulation frequencies, and 2*f*_1_, 2*f*_2_ … 2*f*_n_ represent the corresponding second harmonic positions in frequency domain. FFT: Fast Fourier Transform; LIA: Lock-in Amplifier.

To extend the technique to a practical versatile gas sensor, capable of simultaneous multi-species sensing, several lasers with different modulation frequencies are combined as one beam to probe the gas molecules at the target area. As shown in Figure 1, the combined light is absorbed by different gas molecules. Harmonics (both *f* and 2*f*) of the species can be distinctly separated in the frequency domain, and be simultaneously picked up by LabVIEW-based digital LIAs. Multi-species concentrations are subsequently demodulated at different demodulation frequencies with a normalizing process.

## 3. Instrumentation

### 3.1. Absorption Transition Selection

Due to the advantages of low transmission loss, intrinsic safety, and a wide operating wavelength range in the near-infrared region, a single-mode fiber (SMF-28e^+^, Corning) is employed to guide the lasers for remote multi-species interrogation. As important hydrocarbons in industry and environment [26,27], methane (CH_4_) and acetylene (C_2_H_2_) are selected to demonstrate the implementation of multi-species sensing.

**Figure 2:**
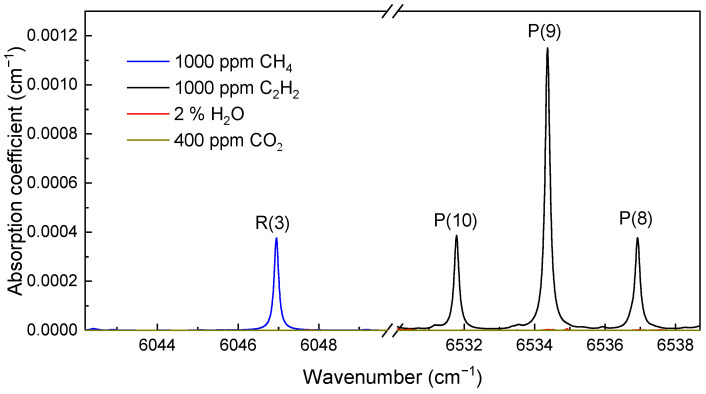
Absorption coefficient simulation for a gas mixture of 1000 ppm CH_4_, 1000 ppm C_2_H_2_, 2% H_2_O and 400 ppm CO_2_ at 296 K and 1 atm.

Figure 2 depicts the calculated absorption spectra of CH_4_ in 6043–6049 cm^−1^ and C_2_H_2_ in 6531.5–6538.5 cm^−1^ based on the HITRAN database [28], showing typical transitions of CH_4_ and C_2_H_2_. The absorption spectrum of 2% H_2_O and 400 ppm CO_2_ are also plotted, considering they are the major interfering components for near-infrared atmospheric sensing. Transitions R(3) at 6046.96 cm^−1^ and P(9) at 6534.36 cm^−1^ are selected as the targets, and they can be easily covered by commercially available laser sources, which are necessary for cost-efficient practical implementation. Influence from atmospheric H_2_O and CO_2_ on CH_4_/C_2_H_2_ sensing would be negligible in cases of the ppm level or higher, such as leakage monitoring.

### 3.2. Experimental Apparatus

**Figure 3:**
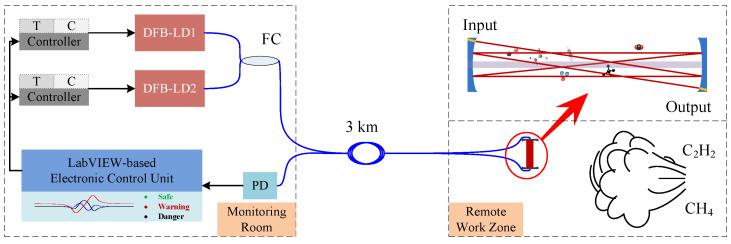
Schematic of the remote sensing system for simultaneous CH_4_ and C_2_H_2_ detection. T, temperature; C, current; DFB, distributed feedback laser diode; FC, fiber coupler; PD, photodetector.

With the optimal target transitions selected, Figure 3 illustrates the sensing configuration for simultaneous CH_4_ and C_2_H_2_ leak detection, gas species identification and potential danger warning. The sensing system comprised the monitoring part and a remote probe, connected by a long conductive fiber. Two distributed feedback (DFB) laser diodes with typical emissions at 1653.7 nm (DFB-LD1) and 1530.4 nm (DFB-LD2) were controlled by a LabVIEW-based electrical control unit via commercial laser diode drivers (LDC501, Stanford Research Systems). Laser current was modulated by applying a sinusoidal dither to the current ramp at different frequencies for CH_4_ and C_2_H_2_. The laser beams were combined as one via a 2 × 1 50:50 fiber coupler and delivered to the work zone via a 3-km optical fiber cable. In the work zone, a custom compact multipass cell was used as a probe, where two quartz mirrors (Φ25.4 mm) coated with dielectric film are 88 mm apart from each other. The beam was introduced and collected by a pair of Φ1.3 mm fiber collimators, leading to a 3-m optical path length after 34 reflections in total. Then the harmonic components were generated by laser-sample interaction and were afterwards demodulated by the monitoring part.

### 3.3. Flow Chart of the Data Retrieval with a LabVIEW-Based Platform

**Figure 4:**
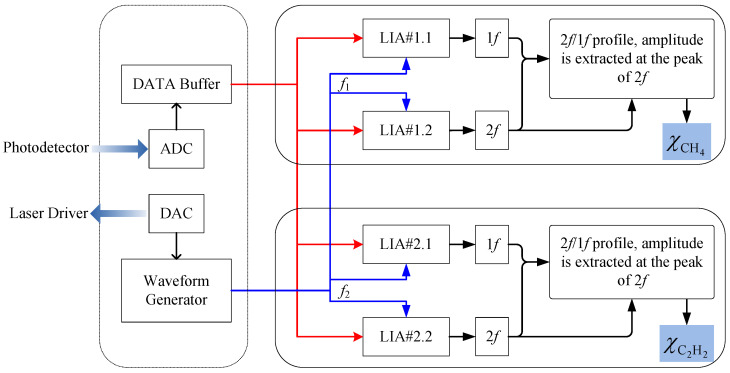
Flow chart of the LabVIEW-based data retrieval. Red lines represent the raw data stream and blue lines represent LIA reference provided by the on-board waveform generator. ADC, analog to digital converter; DAC, digital to analog converter.

Data retrievals were performed using the approach described in Section 2, and the processed spectra data and gas concentrations were related by Equation (10), which was then implemented by a LabVIEW-based platform with its flow chart shown in Figure 4. Transmitted laser signal was acquired by a DAQ card after photoelectric conversion. A waveform generator was used to provide sawtooth signals to modulate DFB lasers with independent dither frequencies. The acquired data was then processed by two couples of LIAs for CH_4_ and C_2_H_2_ respectively.

(10) $$\left\{ \begin{array}{l} S_{1}={\frac{R_{2f}}{R_{1f}}|}_{\omega =2\pi f_{1}}\begin{array}{cc} \to & Gas_{1} \end{array}\\ S_{2}={\frac{R_{2f}}{R_{1f}}|}_{\omega =2\pi f_{2}}\begin{array}{cc} \to & Gas_{2} \end{array}\\ \ldots \\ S_{n}={\frac{R_{2f}}{R_{1f}}|}_{\omega =2\pi f_{n}}\begin{array}{cc} \to & Gas_{n} \end{array} \end{array}\right.$$

LIA#1.2 was used to extract the second harmonic signal of the raw data at a reference frequency of *f*_1_, which was generated by the waveform generator. The 2*f*/1*f* profile was obtained by dividing the magnitude of the first harmonic signal, i.e., output of LIA#1.1, into the second harmonic magnitude. Note that the 1*f* and 2*f* share the same abscissa due to the synchronous trigger. The 2*f*/1*f* amplitude was extracted at the peak position of the 2*f* profile, corresponding to the absorption transition, for the following CH_4_ concentration demodulation. Similarly, LIA#2.1 and LIA#2.2 were used for C_2_H_2_ concentration demodulation at a reference frequency of *f*_2_.

## 4. Experimental Results

**Figure 5:**
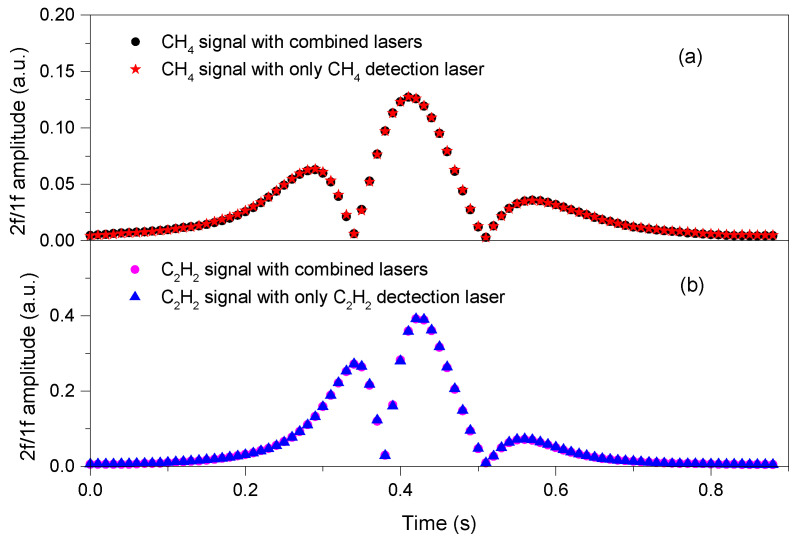
2*f*/1*f* spectral profiles for a mixture of (**a**) 500-ppm CH_4_ and (**b**) 500-ppm C_2_H_2_ diluted in N_2_ at 1 atm and room temperature, with DFB-LD1 modulated at 3 kHz and DFB-LD2 modulated at 4 kHz.

DFB-LD1 and DFB-LD2 were modulated at 3 kHz and 4 kHz respectively, thus CH_4_ and C_2_H_2_ measurements could be operated independently without cross-interference. To characterize the sensor, the probe, together with 500-ppm CH_4_ and 500-ppm C_2_H_2_ diluted in N_2_ was enclosed inside a gas cell. The 2*f*/1*f* spectral scans obtained from the detector signal for three different conditions are illustrated in Figure 5. Figure 5a depicts the detected CH_4_ signals by the first couple of LIAs when both lasers operate and when only the DFB-LD1 operates. The well-matched profiles indicate that simultaneous illumination of both laser beams on the same photodetector does not influence the CH_4_ detection. For C_2_H_2_ detection, a similar conclusion can also be obtained by the signals measured by the second couple of LIAs, shown in Figure 5b. Therefore, both the CH_4_ and C_2_H_2_ can be demodulated separately when both lasers operate simultaneously.

**Figure 6:**
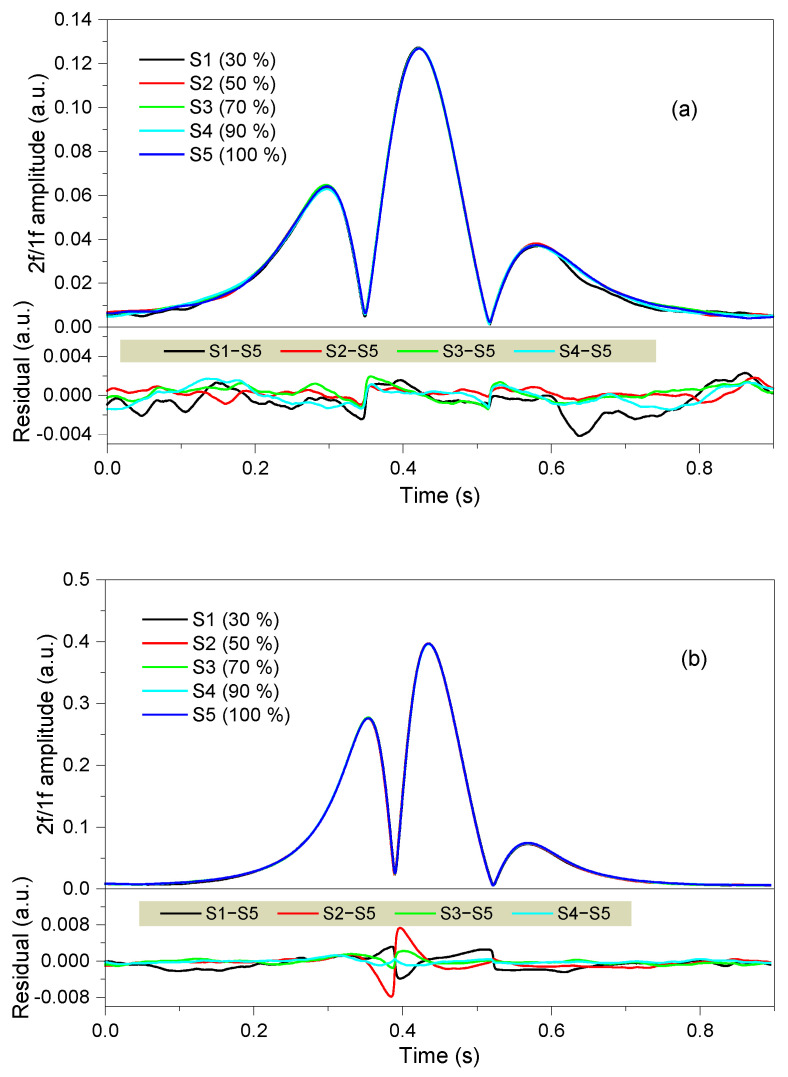
2*f*/1*f* spectral profiles with different laser intensities coupled on the same photodetector for (**a**) 500-ppm CH_4_ and (**b**) 500-ppm C_2_H_2_ diluted in N_2__._

According to Equation (9), the demodulated signal does not relate to the laser intensity. To demonstrate the sensor’s immunity to the laser intensity, an experiment was performed with standard sample gases inside the chamber. The laser intensity was attenuated from its maximum down to 90%, 70%, 50%, and 30% respectively. Original data were acquired at a sampling rate of 200 kS/s, and the following process was performed according to the flow chart in Figure 4. It took 1 s for a single measurement, 0.9 s for data acquisition and 0.1 s for data processing. The measured 2*f*/1*f* profiles are illustrated in Figure 6. From the profiles of 500-ppm CH_4_ shown in Figure 6a, the 2*f*/1*f* waveforms keep stable with a wide intensity range. The worst correlation coefficient compared to the profile at maximum intensity occurs when the laser intensity is attenuated to 30%, but is still better than 0.9992. Meanwhile, the correlation coefficients of the 500-ppm C_2_H_2_ profiles, shown in Figure 6b, are calculated to be better than 0.9998.

**Figure 7:**
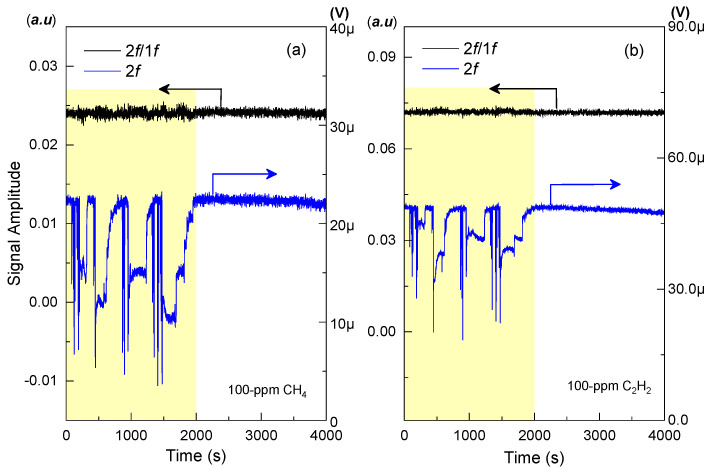
Long-term monitoring of sample gas mixture sealed in the chamber, illustrating a comparison between 2*f*/1*f* results and 2*f* results for both (**a**) 100-ppm CH_4_ and (**b**) 100-ppm C_2_H_2_. The left axes pointed by the black arrows mean the amplitude of 2*f*/1*f* signal, and the right axes pointed by the blue arrows mean the amplitude of 2*f* signal.

Besides the test with fixed attenuation, its immunity to dynamic laser intensity variation was further investigated by irregularly attenuating the laser beams. With a mixture of 100-ppm CH_4_ and 100-ppm C_2_H_2_ filled in the chamber, the investigation was performed over 4000 s with the results shown in Figure 7. The first half of the data with a pale-yellow background was obtained at the condition of drastically intensity variation with an irregular attenuation ranging from 0 to 6 dB, while the second half corresponds to a stable one. From Figure 7a, it can be seen that the measured CH_4_ data with normalizing process, i.e., 2*f*/1*f*, keep rather stable compared to the conventional 2*f* signal for gas analysis, and its standard deviation with irregular variation is calculated to be only 1.38 times as much as that with a stable laser intensity. From the C_2_H_2_ data in Figure 7b, this parameter is calculated to be only 1.23. Therefore, the designed sensor would demonstrate a high immunity to the laser variation coupled on the photodetector in practical application.

**Figure 8:**
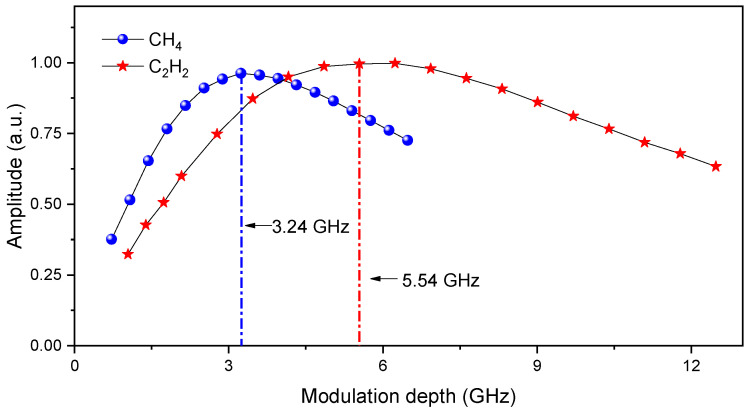
Normalized signal amplitude as a function of modulation depth.

Due to the line-width of an absorption transition, the harmonic signals can be influenced by the modulation depth in its amplitude and spectral waveform. The dependence of 2*f*/1*f* signal amplitude as a function of the modulation depth was experimentally investigated with the results depicted in Figure 8. The responses for CH_4_ and C_2_H_2_ measurements are plotted in blue dots and red stars respectively. The 2*f*/1*f* signal for CH_4_ increases with modulation depth, but no further increase was observed when the modulation depth is larger than 3.24 GHz. Similarly, the optimum modulation depth for C_2_H_2_ was 5.54 GHz. Hence, the modulation depths of 3.24 GHz and 5.54 GHz were selected in the following work to obtain the maximum signals.

**Figure 9:**
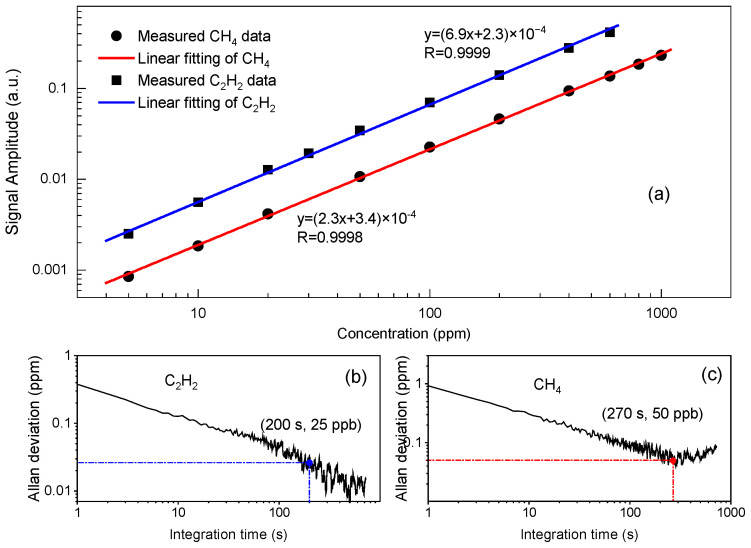
(**a**) Signal amplitude as a function of sample gas concentration; Allan deviation analysis as a function of integration time for (**b**) C_2_H_2_ and (**c**) CH_4_. The sensor shows a good linear response for both C_2_H_2_ and CH_4_. The MDLs at 1 s are 0.4 ppm and 1 ppm for C_2_H_2_ and CH_4_ respectively. An MDL of 25 ppb for C_2_H_2_ at 200 s and 50 ppb for CH_4_ at 270 s can be obtained.

With the optimized modulation depths, the sensor performance was further investigated by measuring a series of CH_4_/C_2_H_2_/N_2_ mixtures with known concentrations. The mixtures were diluted with N_2_ using three mass flow meters to different mixing ratios within 1000 ppm. The measured signal amplitude as a function of sample gas concentration is depicted in Figure 9a with circle (CH_4_) and square (C_2_H_2_) dots. The calculated R-square values are larger than 0.9998, indicating a good approximation of the linear fitting line to the real data points. This implies that the sensor system exhibits an excellent linear response to both CH_4_ and C_2_H_2_ at atmospheric pressure.

To evaluate the long-term stability and detection limit of the sensor, an Allan deviation analysis was performed by measuring the signal amplitudes of a gas mixture, comprising 100-ppm CH_4_ and 20-ppm C_2_H_2_, for one hour. The time resolution of the data acquisition was set to 1 s. Detection limits of the sensor could be further evaluated by the obtained fitting equations in Figure 9a. Figure 9b,c, report the Allan deviation with a minimum detection limit (MDL) of 0.4 ppm for C_2_H_2_ and 1 ppm for CH_4_ respectively at 1 s integration time. Furthermore, an MDL of 25 ppb for C_2_H_2_ at 200 s and 50 ppb for CH_4_ at 270 s respectively was reported, exhibiting good stability for the current system.

**Figure 10:**
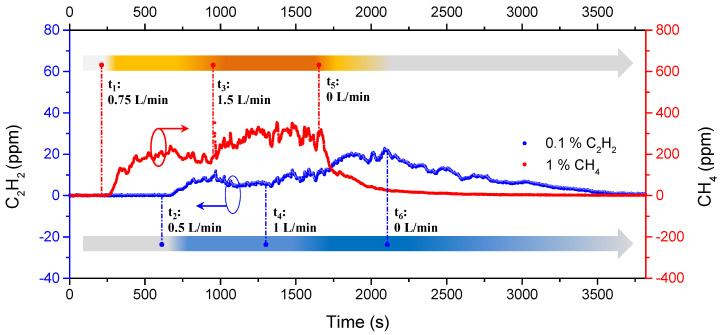
Time history curves of recorded CH_4_ and C_2_H_2_ concentration at different leakage conditions. t_1_ to t_6_ represent different leakage rates at different times, and the leakage rate can be controlled by two mass flow meters. The arrows in yellow and blue illustrate the measured relative leakage over time and the dark color means more leakage than light color.

After characterization and calibration with the above experiments, the open-ended probe was installed in a work zone to verify its capability of monitoring hydrocarbon leakage. With a 3 km optical fiber cable, the other parts, except the sensing probe, were operated in a monitoring room at 23 ± 1 °C temperature and 50 ± 10% RH, which is a moderate operation condition for the DFB lasers and most other electronic equipment. The hydrocarbon leaked from two gas cylinders, 1% CH_4_ and 0.1% C_2_H_2_, were both diluted in N_2_. The maximum concentrations are much lower than the explosion limits, i.e., 5% for CH_4_ and 2.3% for C_2_H_2_, for security. By employing two mass flow meters, the CH_4_ leakage was actively controlled at 0.75 L/min from t_1_, 1.5 L/min from t_3_, then was closed at t_5_. For C_2_H_2_, the leakage was 0.5 L/min from t_2_ and rose to 1 L/min from t_4_, then was closed at t_6_. Figure 10 illustrates the recorded time history curves with different leakage times, different leakage rates, and different gas species. We observed the CH_4_ at around 200 ppm after t_1_, then an apparent increase to about 300 ppm after t_3_, finally a decline from t_5_. Similarly, the C_2_H_2_ was observed at about 10 ppm after t_2_, an ascent to about 20 ppm after t_4_, then a gradual decline from t_6_. The curves’ tendency matches the leakage conditions well, indicating its high practicability in real-time remote multi-species monitoring applications.

## 5. Conclusions

In summary, we developed a practical spectroscopic sensor combining normalized wavelength modulation spectroscopy and frequency-division multiplexing for real-time multi-species monitoring. The first harmonic signal was employed to suppress the intensity noise in order to improve the immunity to irregular laser intensity noise. Different gas species in the sensing area can be remotely demodulated at separate frequencies without any cross-interference. A centimeter-size multi-pass cell with a 3-m optical length was developed as a compact terminal probe. CH_4_ and C_2_H_2_ were chosen to demonstrate the sensor’s characterization and performance. Two DFB laser diodes, emitting at 1653.7 nm and 1530.4 nm, were independently modulated at 3 kHz and 4 kHz to target the R(3) transition of CH_4_ and P(9) transition of C_2_H_2_ respectively. After optimizing the wavelength modulation depth, the combined laser beam can simultaneously interrogate the CH_4_ and C_2_H_2_ with sub-ppm MDLs with 1 s time resolution. Furthermore, an application test for remote hydrocarbon leakage monitoring was carried out with a good match between the recorded results and the controllable leakage conditions, indicating promising and reliable remote multi-species sensing in real-world applications. Besides, since the sensing system operates with silica-based single-mode fiber in near-infrared, covering absorption transitions of many different gas species [28], the current sensor can be easily extended to monitor more industrial hazardous gas species without scarifying the sensing performance.

## Data Availability

The data that support the plots within this paper are available from the corresponding author on request basis.
